# Terpenes as Potential Multi-Target Modulators of Chronic Stress-Induced Neuroendocrine–Immune Dysregulation

**DOI:** 10.3390/ijms27167242

**Published:** 2026-08-14

**Authors:** Dušica M. Kočović, Sanja Momčilović, Tamara Svetozarević

**Affiliations:** Group for Neuroendocrinology, Institute for Medical Research, National Institute of Republic of Serbia, University of Belgrade, 11129 Belgrade, Serbia

**Keywords:** chronic stress, HPA axis, neuroendocrine–immune dysregulation, terpenes, stress-related disorders

## Abstract

Chronic stress is increasingly recognized as a major contributor to systemic disease. Prolonged or repeated activation of the hypothalamic–pituitary–adrenal (HPA) axis and sympathoadrenal systems may contribute to glucocorticoid receptor resistance and chronic low-grade inflammation, although stress-related responses vary depending on stressor characteristics, population, age, sex, health status, and study design. Elevated levels of pro-inflammatory cytokines (IL-6, IL-1β, TNF-α), oxidative stress, and NF-κB activation contribute to immune dysfunction, neuroinflammation, metabolic disturbances, reproductive and endocrine imbalance, as well as structural and functional alterations in stress-sensitive brain regions. Prolonged exposure to stress may contribute to the development of neuropsychiatric, cardiovascular, metabolic, neurodegenerative, and malignant diseases, highlighting the need for effective strategies to mitigate the detrimental consequences of chronic stress. Given the limitations and adverse effects associated with conventional pharmacological approaches, increasing attention has been directed toward natural bioactive compounds. Terpenes represent a diverse class of phytochemicals with reported anti-inflammatory, antioxidant, and neuromodulatory properties. Terpenes such as limonene and β-caryophyllene, as well as terpenoids such as linalool, may modulate NF-κB, MAPK, and Nrf2 signaling pathways and interact with the endocannabinoid system, thereby attenuating neuroinflammation, oxidative stress, and stress-induced immune dysregulation. This review summarizes current evidence regarding the mechanisms linking chronic stress, HPA axis dysfunction, and systemic disease, with particular emphasis on endocrine, immune, and nervous system alterations. In addition, it critically evaluates the mechanistic and translational potential of terpenes as possible indirect neuroimmune and redox modulators of stress-related pathophysiology.

## 1. Introduction

### 1.1. Chronic Stress and HPA Axis Dysregulation

Chronic stress results from prolonged exposure to stressors that exceed the organism’s adaptive capacity, leading to persistent physiological and psychological alterations. Although stress is an integral component of physiological adaptation, its biological impact largely depends on duration and intensity. In contrast to acute stress, which elicits transient adaptive responses essential for survival, prolonged stress exposure promotes sustained neuroendocrine activation, disruption of homeostasis, and progressive physiological and psychological dysfunction. Modern lifestyle factors, including sedentary behavior, unhealthy dietary habits, and prolonged psychological stress, further exacerbate stress-related symptoms such as anxiety, depression, insomnia, irritability, and mental fatigue [1]. Consequently, prolonged stress exposure has been associated with increased vulnerability to numerous pathological conditions, including cancer, diabetes, and cardiovascular, respiratory, gastrointestinal, immunological, and neurological disorders [2,3,4,5,6].

### 1.2. Physiology of HPA Axis

The hypothalamic–pituitary–adrenal (HPA) axis represents the principal neuroendocrine system coordinating physiological responses to stress [7]. It functions as an integrated neuroendocrine communication network linking the central nervous system with peripheral endocrine organs. Stress-related signals activate neurons in the hypothalamic paraventricular nucleus (PVN), leading to the release of corticotropin-releasing hormone (CRH) and vasopressin (AVP), which stimulate the secretion of adrenocorticotropic hormone (ACTH) from the anterior pituitary gland. ACTH subsequently acts on the adrenal cortex to induce the synthesis and release of glucocorticoids, primarily cortisol in humans [7]. Cortisol plays a central role in energy mobilization, immune regulation, and maintenance of systemic homeostasis during stress exposure. In the central nervous system, cortisol influences neuronal survival, neurogenesis, memory formation, and emotional processing, highlighting its critical role in brain function [8]. To prevent excessive or prolonged activation, the HPA axis is tightly regulated through a negative feedback mechanism. Circulating cortisol suppresses CRH and ACTH release at the hypothalamus and pituitary levels, primarily via glucocorticoid and mineralocorticoid receptors expressed in stress-sensitive brain regions and endocrine tissues [9].

### 1.3. HPA Axis in Pathology

**Figure 1:**
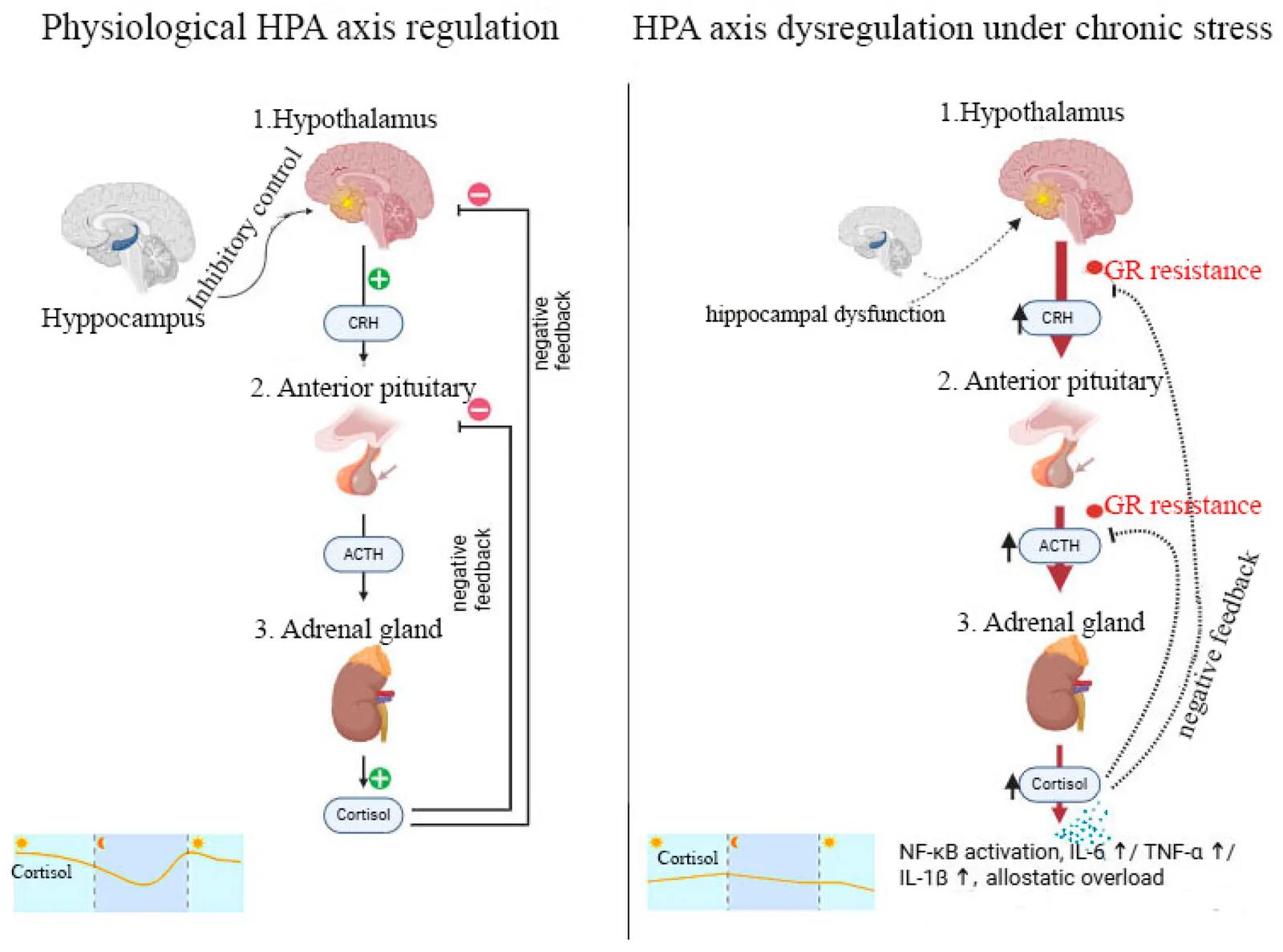
Transition from adaptive HPA axis regulation to chronic stress-induced neuroendocrine dysregulation. The figure illustrates activation of the hypothalamic–pituitary–adrenal (HPA) axis under physiological conditions (**left panel**), with hypothalamic corticotropin-releasing hormone (CRH) signaling, pituitary adrenocorticotropic hormone (ACTH) release, and adrenal cortisol secretion regulated by glucocorticoid-mediated negative feedback at the hypothalamus and pituitary, with additional hippocampal inhibitory control, maintaining circadian cortisol rhythmicity and systemic homeostasis. The (**right panel**) depicts HPA axis dysregulation under chronic stress, where prolonged glucocorticoid exposure impairs negative feedback sensitivity, promotes glucocorticoid receptor resistance, and disrupts hippocampal inhibitory control, resulting in flattened or dysregulated cortisol rhythms. Sustained HPA axis activation is associated with increased inflammatory signaling, including nuclear factor kappa B (NF-κB) activation and elevated pro-inflammatory cytokines (IL-1β, IL-6, and TNF-α), contributing to a maladaptive neuroendocrine–immune feedback loop and allostatic overload.

Whereas acute stress response is predominantly adaptive, persistent HPA activation disrupts feedback regulation, resulting in persistent glucocorticoid dysregulation and cortisol abnormalities that promote inflammation and constitute a key pathological mechanism underlying chronic stress-related disorders [7,10]. Sustained glucocorticoid exposure progressively impairs negative feedback regulation at hippocampal, hypothalamic, and pituitary levels, thereby reinforcing neuroendocrine dysregulation and allostatic overload [9]. Key mechanisms underlying the transition from adaptive HPA axis regulation to chronic stress-associated dysregulation are summarized in Figure 1.

Chronic HPA axis dysfunction has been implicated in the pathogenesis of neuropsychiatric disorders, including major depressive disorder, anxiety disorders, and post-traumatic stress disorder, as well as metabolic, cardiovascular, and immune-mediated inflammatory diseases [11,12]. Persistent HPA axis overactivation may also contribute to neurodegenerative processes by promoting hippocampal atrophy, oxidative stress, and neuroinflammation, thereby exacerbating neuronal vulnerability in disorders such as Alzheimer’s and Parkinson’s disease [13]. Furthermore, HPA axis dysregulation has been linked to cancer, including breast cancer, where elevated glucocorticoid levels can suppress antitumor immunity, enhance pro-tumorigenic inflammation, and promote tumor growth and metastasis [14]. Disrupted cortisol rhythms associated with chronic stress may further impair immune surveillance and contribute to poorer clinical outcomes in cancer patients.

### 1.4. Terpenes as Modulators of HPA Axis Dysfunction

Given the central role of HPA axis dysregulation in chronic stress and stress-related diseases, restoration of neuroendocrine homeostasis represents a critical therapeutic objective. Conventional pharmacological interventions, including glucocorticoid receptor antagonists, selective serotonin reuptake inhibitors (SSRIs), and benzodiazepines, are effective for symptom management but are frequently limited by adverse effects such as immunosuppression, metabolic disturbances, sedation, tolerance, and incomplete normalization of HPA axis function [15].

In recent years, natural bioactive compounds, particularly terpenes, have gained attention as potential complementary modulators of stress-related pathophysiology [16]. Terpenes exhibit pleiotropic biological effects, including attenuation of neuroinflammation, antioxidant effects, immune modulation, and neuroprotection, all of which are closely linked to chronic HPA axis activation and stress-associated neuroendocrine perturbations [17]. Preclinical studies demonstrate that specific monoterpenes, such as α-pinene and β-pinene, exert neuroprotective effects in cellular models of neurodegeneration, including reduction in amyloid-β-induced toxicity and inhibition of amyloid fibril formation, processes relevant to Alzheimer’s disease pathology [17].

In experimental models, isolated terpenes and selected compounds belonging to the modified class of terpenes known as terpenoids have been reported to modulate inflammatory and redox-sensitive pathways by suppressing pro-inflammatory cytokines, inhibiting NF-κB signaling, and reducing oxidative stress biomarkers. These mechanisms may be relevant to chronic stress-associated neuroinflammation and immune dysregulation [18,19]. These findings support mechanistic relevance to chronic stress-associated neuroinflammation and immune dysregulation, but should not be interpreted as direct evidence of endocrine regulation in humans [18,19]. Broad mechanistic and clinical evidence for terpenes directly altering HPA axis hormones, such as ACTH or cortisol, remains limited. Most of the available data come from studies of essential oils, complex mixtures of aromatic volatile compounds in which terpenes are the predominant constituents, and from preclinical models, rather than from controlled human trials using isolated compounds [20,21]. Nevertheless, reviews of individual terpenes, such as α-pinene, and terpenoids, such as linalool, report neuroactive and stress-relevant effects and highlight the need for further investigation into their potential endocrine actions [17,22,23].

In oncology-related models, the anti-inflammatory and antioxidant properties of terpenes/terpenoids intersect with key aspects of tumor biology by modulating immune surveillance, apoptotic pathways, and pro-tumorigenic inflammatory signaling within the tumor microenvironment [24]. Terpenoids have been shown to induce cell-cycle arrest and apoptosis via regulation of p53, Bax/Bcl-2 family proteins, caspase activation, and ROS-mediated signaling, while concurrently downregulating survival pathways such as PI3K/Akt/mTOR and JAK/STAT. Moreover, these compounds can enhance antitumor immune responses and improve the efficacy of conventional therapies by modulating immune effector mechanisms and reducing tumor-associated inflammation [24].

**Figure 2:**
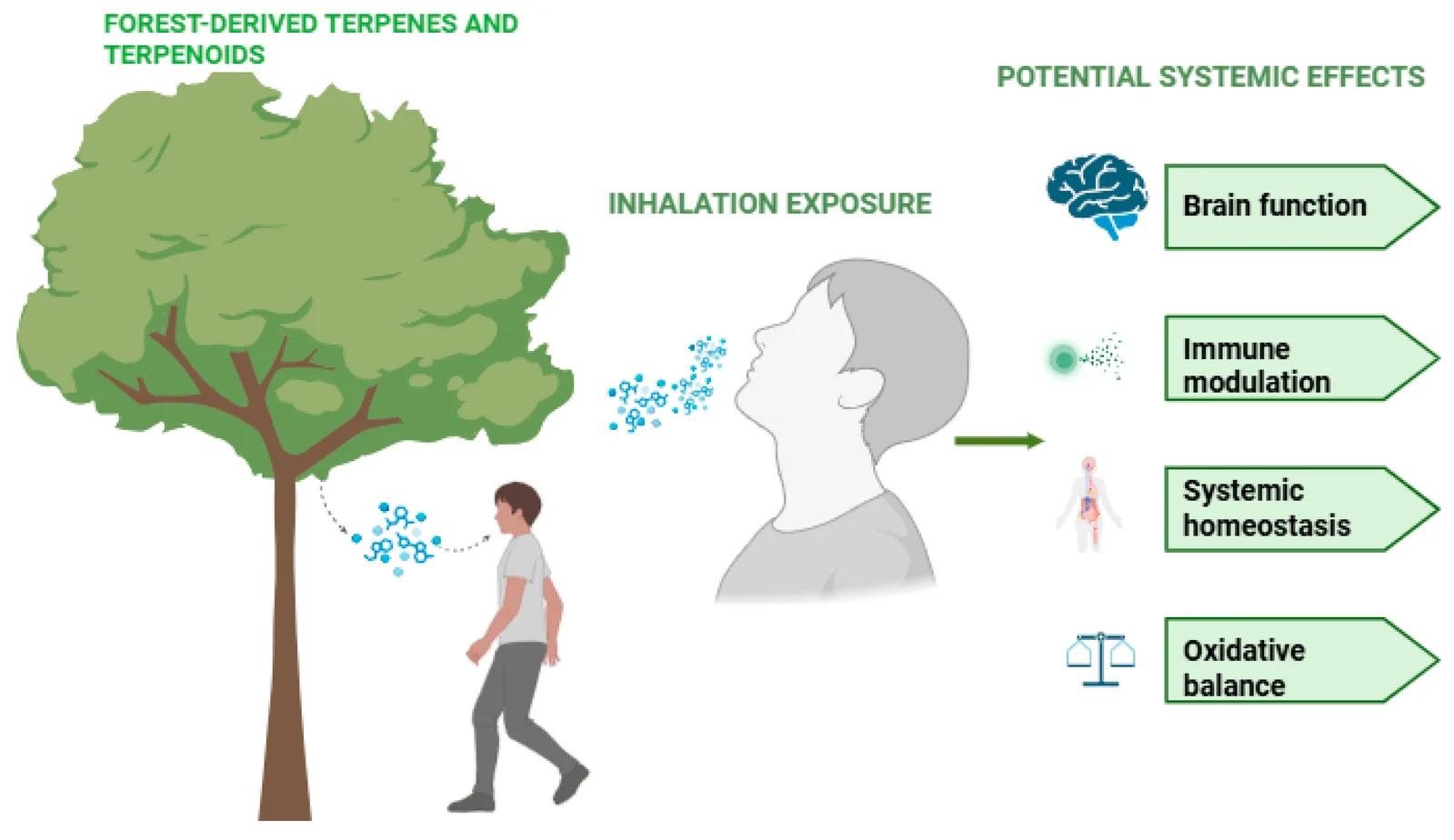
Conceptual overview of environmental exposure to forest-derived terpenes and terpenoids and their potential systemic effects on stress-responsive physiological systems. Terpenes and terpenoids constitute the major biogenic volatile compounds in forest aerosols. Upon inhalation via the respiratory tract, they may modulate nervous, immune, and systemic pathways that contribute to stress regulation, inflammatory responses, oxidative balance, and the maintenance of homeostasis.

Taken together, this heterogeneous evidence supports the hypothesis that terpene-related exposures may act as potential multi-target modulators of stress-responsive neuroimmune and redox pathways, although the strength and specificity of the evidence vary substantially across isolated compounds, essential oils, phytoncide exposure, and experimental models. Beyond experimental and clinical models, these mechanistic insights are reflected in real-world interventions such as forest bathing (Shinrin-yoku), a structured form of nature-based therapy shown to promote physical and psychological well-being [25]. The benefits of forest exposure have been attributed in part to inhalation of terpenes and terpenoids, and other biogenic volatile compounds emitted by trees, providing a biologically plausible link between environmental exposure and molecular pathways involved in stress regulation. However, forest-bathing studies involve complex environmental exposures that include physical activity, visual and sensory stimulation, psychological relaxation, and social or contextual factors, in addition to inhalation of biogenic volatile organic compounds. Therefore, these studies were interpreted separately from interventions using isolated terpenes or defined essential oil preparations. Historically, the health-promoting effects of natural environments were recognized by Hippocrates and later conceptualized as Vis Medicatrix Naturae. Contemporary research increasingly substantiates these early observations by identifying specific plant-derived compounds capable of modulating stress-related biological pathways. Figure 2 illustrates the potential systemic effects of inhaled forest-derived terpenes on the brain, immune system, oxidative balance, and overall homeostasis.

Within this framework, terpenes emerge as chemically defined compounds with potentially tractable mechanisms that may bridge empirical observations of nature-based therapies with modern neuroendocrine and immunopharmacology, highlighting their potential relevance to strategies aimed at mitigating chronic stress and HPA axis dysfunction. The following sections summarize the impact of chronic stress and HPA axis dysregulation on endocrine, immune, and nervous systems, followed by a critical overview of current mechanistic and translational evidence supporting terpenes as modulators of stress-associated pathophysiology.

### 1.5. Literature Search and Methodology

This article was designed as a narrative review. A literature search was performed using PubMed/MEDLINE, Scopus, Web of Science, and Google Scholar for publications available up to 20 May 2026, with priority given to peer-reviewed articles published within the last 10 years. Seminal older studies were included when they provided foundational concepts related to HPA axis physiology, chronic stress biology, glucocorticoid receptor resistance, stress immunology, neuroinflammation, or terpene pharmacology. Search terms included combinations of “chronic stress”, “HPA axis”, “sympathoadrenal system”, “glucocorticoids”, “glucocorticoid receptor”, “DHEA”, “neuroendocrine immune dysregulation”, “cytokines”, “NF-kB”, “oxidative stress”, “neuroinflammation”, “microglia”, “terpenes”, “terpenoids”, “essential oils”, “linalool”, “limonene”, “α-pinene”, “β-caryophyllene”, “1,8-cineole”, and “stress-related disorders”. The search strategy combined stress-related, neuroendocrine, immune, redox, and terpene-related terms to identify studies relevant to the scope of this narrative review.

Eligible articles included original experimental studies, clinical or translational studies, and relevant reviews addressing chronic stress-induced endocrine, immune, metabolic, hematopoietic, or neural dysregulation, as well as biological effects of terpenes relevant to inflammation, oxidative stress, neuroprotection, immune regulation, or stress-related behavioral outcomes. Articles were excluded if they were not peer-reviewed, not available in English, unrelated to the scope of the review, or focused exclusively on terpene isolation, structural characterization, or analytical chemistry without biological or stress-related relevance. As this was a narrative review, no formal meta-analysis was performed; the literature was selected and prioritized based on relevance, mechanistic depth, recency, translational significance, and consistency with the review framework.

Given the heterogeneity of the available evidence, the literature was interpreted according to the type of terpene-related exposure and the level of experimental or clinical evidence. Throughout the review, we distinguished isolated terpene compounds, such as linalool, limonene, α-pinene, 1,8-cineole, and β-caryophyllene, and chemically broader terpenoid compounds such as linalool and 1,8-cineole, from complex essential oil preparations. Studies using essential oils were considered separately from studies of phytoncide or forest-derived volatile exposure, because the latter may include environmental, behavioral, and nature-exposure components in addition to inhaled volatile organic compounds. Evidence was also stratified according to study type, including experiments in vitro, animal studies, human observational or interventional studies, and disease models not specifically designed as chronic stress models. Findings from in vitro studies and disease models not specifically designed to investigate chronic stress were used primarily to identify relevant mechanisms, whereas human studies and chronic stress models were prioritized when evaluating stress-related physiological relevance. This evidence stratification is summarized in Supplementary Table S1.

## 2. Chronic Stress-Induced Endocrine, Immune, and Neural Dysregulation

### 2.1. Endocrine Consequences of Chronic Stress

Prolonged stress exposure disrupts endocrine homeostasis through sustained activation of the HPA axis and its interactions with the hypothalamic–pituitary–gonadal, hypothalamic–pituitary–thyroid, growth hormone/IGF-1, metabolic, and sympathoadrenal systems. Although stress-responsive endocrine signaling is essential for adaptive regulation of metabolism, cardiovascular function, immunity, and behavior, persistent activation promotes systemic dysregulation and increases vulnerability to stress-associated disorders [26].

#### 2.1.1. Cortisol in Chronic Stress-Induced HPA Axis Dysregulation

Cortisol is the principal glucocorticoid and the primary hormonal effector of the HPA axis. Under physiological conditions, cortisol secretion follows a tightly regulated circadian rhythm, with peak concentrations in the early morning and lower levels during the evening and night. In response to stress, the hypothalamus releases corticotropin-releasing hormone (CRH) and arginine vasopressin. These stimulate pituitary secretion of adrenocorticotropic hormone (ACTH), which in turn drives cortisol synthesis and release from the adrenal cortex. Cortisol facilitates adaptation to stress by increasing glucose availability while regulating cardiovascular tone, cognition, arousal, and inflammatory responses. Persistent or repeated HPA axis activation may result in heterogeneous cortisol patterns. Depending on the clinical context, HPA axis dysregulation may manifest as hypercortisolism, flattened diurnal cortisol rhythms, altered cortisol awakening responses, exaggerated or blunted stress reactivity, or, in some conditions, hypocortisolism. These heterogeneous patterns suggest broader disturbances in dynamic HPA axis regulation, indicating that endocrine responses to chronic stress are not uniform. Sustained glucocorticoid exposure progressively impairs negative feedback sensitivity at hippocampal, hypothalamic, and pituitary levels. Over time, this contributes to allostatic overload [26,27].

In some chronic, stress-related contexts, prolonged or repeated stress exposure has been associated with reduced glucocorticoid sensitivity or glucocorticoid receptor (GR) resistance. Under conditions of glucocorticoid receptor resistance, immune cells become less sensitive to the anti-inflammatory actions of cortisol despite ongoing glucocorticoid exposure. As a result, cortisol-mediated suppression of inflammatory transcriptional programs becomes impaired, allowing persistent activation of pro-inflammatory pathways including NF-κB and sustained production of cytokines such as IL-1β, IL-6, and TNF-α. Consequently, prolonged stress exposure may paradoxically coexist with both sustained glucocorticoid signaling and chronic low-grade inflammation [28].

#### 2.1.2. Testosterone Under Chronic Stress

The HPA axis interacts closely with the hypothalamic–pituitary–gonadal (HPG) axis. Under prolonged stress exposure, CRH, glucocorticoids, catecholamines, and inflammatory cytokines disrupt HPG axis function at multiple levels. Persistent HPA axis activation suppresses gonadotropin-releasing hormone (GnRH), luteinizing hormone (LH), and follicle-stimulating hormone (FSH) signaling, thereby impairing gonadal steroidogenesis. In males, this may contribute to reduced testosterone production, altered spermatogenesis, and impaired reproductive function [29]. Testosterone also has important immunomodulatory and metabolic effects. Reduced androgen signaling during chronic stress may influence body composition, energy metabolism, mood, motivation, and inflammatory balance. In stress-related inflammatory conditions, low androgen levels may attenuate the anti-inflammatory and anabolic actions associated with testosterone. Overall, testosterone is considered part of an integrated neuroendocrine–immune crosstalk through which chronic stress may influence reproductive and systemic physiological function [29,30].

#### 2.1.3. DHEA and Adrenal Androgen Balance in Chronic Stress

In addition to cortisol, the adrenal cortex produces adrenal androgens, particularly dehydroepiandrosterone (DHEA) and its sulfate ester DHEA-S. DHEA and DHEA-S may counterbalance glucocorticoid-mediated effects through actions on immune, neural, and metabolic pathways. Under chronic stress, the balance between cortisol and DHEA/DHEA-S may be altered. The cortisol/DHEA or cortisol/DHEA-S ratio is often considered an indicator of the relative catabolic versus anabolic balance of the endocrine system [31]. An increased cortisol/DHEA ratio may reflect reduced anabolic and stress-buffering capacity and has been associated with impaired immune regulation and metabolic vulnerability, although findings vary according to age, sex, stressor type, disease state, and study design [31,32]. Alterations in cortisol/DHEA balance may therefore represent an additional mechanism linking prolonged stress exposure with neuroimmune dysfunction. However, the clinical interpretation of cortisol/DHEA or cortisol/DHEA-S ratios requires caution, as these measures are influenced by circadian rhythm, age, sex, health status and medication use.

#### 2.1.4. Estrogen, Progesterone and Female Reproductive Function Under Chronic Stress

In females, chronic stress may disrupt ovarian function through HPA-mediated suppression of the HPG axis. Prolonged stress-induced activation of CRH and cortisol signaling may disrupt pulsatile GnRH secretion and disturb LH and FSH release, thereby altering follicular development, ovulation, and corpus luteum function. As a result, chronic stress has been associated with irregular ovulation, anovulation, luteal phase dysfunction, reduced fertility, and functional hypothalamic amenorrhea [33,34]. Estrogens and progesterone also regulate stress responsiveness. Estrogen can modulate HPA axis activity, glucocorticoid receptor signaling, monoaminergic neurotransmission, and immune function, whereas progesterone and its neuroactive metabolites, particularly allopregnanolone, influence GABAergic signaling and stress-related emotional regulation. These interactions reflect reciprocal regulation between ovarian hormones and HPA axis activity [34,35]. Altered estrogen and progesterone signaling may contribute to differences in stress responsivity, affective and anxiety disorders, and reproductive dysfunction. The effects of stress may vary according to menstrual cycle phase, pregnancy, postpartum state, perimenopause and/or menopause, indicating that endocrine context is a critical determinant of stress responsivity [35].

#### 2.1.5. Thyroid Hormones, Prolactin, Growth Hormones and Metabolic Regulation in Response to Chronic Stress

Chronic stress may also affect the hypothalamic–pituitary–thyroid (HPT) axis. Stress-related glucocorticoid and cytokine signaling can influence thyrotropin-releasing hormone (TRH), thyroid-stimulating hormone (TSH), peripheral conversion of thyroxine to triiodothyronine, as well as tissue sensitivity to thyroid hormones. However, findings regarding thyroid hormone alterations remain inconsistent and appear highly context-dependent [36].

Prolactin and growth hormone may also change during stress exposure. Whereas acute stress transiently increases prolactin and GH secretion, prolonged stress exposure produces heterogeneous endocrine responses. Both prolactin and GH interact with immune regulation, metabolism, tissue repair, and may partly counterbalance the immunosuppressive and catabolic effects of prolonged glucocorticoid exposure. These hormonal alterations contribute to broader endocrine–immune and metabolic dysregulation [36,37]. Metabolic regulation is also affected by chronic stress. Persistent glucocorticoid exposure impairs glucose homeostasis and promotes insulin resistance, visceral adiposity, dyslipidemia, and altered appetite-related signaling. Through these mechanisms, chronic stress may increase vulnerability to metabolic syndrome, type 2 diabetes, and cardiovascular disease [26].

#### 2.1.6. Catecholamines and Sympathoadrenal Activation Under Chronic Stress

Although cortisol is central to the endocrine stress response, chronic stress also involves repeated or prolonged activation of the sympathoadrenal medullary system, resulting in increased adrenergic signaling and release of catecholamines, primarily adrenaline and noradrenaline. Catecholamines support acute adaptation by increasing cardiovascular activity, glucose availability, and leukocyte redistribution. However, when sympathoadrenal activation is sustained or repeatedly triggered, prolonged catecholamine exposure may contribute to hypertension, endothelial dysfunction, oxidative stress, altered leukocyte trafficking, and persistent inflammatory activation. Through adrenergic receptor signaling, catecholamines modulate cytokine production, leukocyte trafficking, and inflammatory gene expression, thereby linking chronic stress with cardiovascular, immune, and metabolic dysregulation [28]. Collectively, prolonged stress exposure disrupts endocrine homeostasis through bidirectional interactions among neuroendocrine, metabolic, and immune pathways. These alterations affect immune regulation, neuroinflammation, energy metabolism, reproductive function, cognition, and emotional behavior, establishing endocrine dysregulation as a central mechanistic link between chronic stress and systemic disease. This framework further provides a biologically relevant context for evaluating terpenes as potential modulators of stress-associated neuroimmune dysfunction.

### 2.2. Chronic HPA/SAM Activation–Immune Dysregulation

Chronic stress and HPA axis dysregulation profoundly affect systemic physiology, with the immune system as a primary target. Prolonged activation of the HPA axis and sympathetic nervous system promotes persistent exposure of immune cells to glucocorticoids and catecholamines, which results in the emergence of glucocorticoid resistance at the cellular level [38]. This is characterized by reduced sensitivity of immune cells to the anti-inflammatory actions of cortisol, due to altered glucocorticoid receptor expression, impaired nuclear translocation, and dysregulated receptor signaling [38,39,40]. Altered GRα/GRβ isoform balance, post-translational receptor modifications, and disrupted co-regulator recruitment contribute to impaired glucocorticoid-mediated transcriptional repression of inflammatory genes. As a consequence, negative regulation of inflammatory gene transcription is diminished, allowing pro-inflammatory pathways to remain chronically active despite elevated circulating cortisol levels. Understanding stress-induced cellular and molecular changes provides a basis for evaluating bioactive compounds, including terpenes, that may help restore immune balance. Chronic stress is associated with reductions in NK cell activity, lymphocyte proliferation, and antibody production. Induced neuroendocrine changes also disrupt leukocyte trafficking, intracellular signaling pathways, and cytokine balance, leading to immunosuppression, persistent low-grade inflammation, and increased risk of stress-related disorders [41,42,43].

#### 2.2.1. Natural Killer (NK) Cells

Natural killer (NK) cells are cytotoxic lymphocytes of the innate immune system that rapidly defend against tumor cells and virus-infected cells in the absence of prior antigen-specific immunization. NK cells act through direct cytotoxicity and the secretion of cytokines, including interferon-γ (IFN-γ), tumor necrosis factor-α (TNF-α), and interleukin-10 (IL-10), as well as growth factors and multiple chemokines [44]. While acute stress can increase circulating NK cell numbers, chronic or repeated stress exposure has often been associated with reduced NK cell number, altered distribution, and impaired function, although the magnitude and direction of these effects may vary across stress models and clinical contexts. Elevated glucocorticoid levels inhibit NK cell cytotoxicity by downregulating perforin and granzyme expression and suppressing IFN-γ production. Additionally, catecholamines interfere with NK cell trafficking, target-cell recognition, and cytotoxic activity [45]. Prolonged glucocorticoid exposure further promotes NK cell apoptosis and reduces responsiveness to activating cytokines such as IL-2 and IL-12 [45]. These alterations compromise antiviral defense and tumor immune surveillance, particularly under chronic or cancer-associated stress conditions.

#### 2.2.2. Macrophages and Neutrophils

Macrophages and neutrophils are key components of the innate immune system, playing essential roles in host defense, inflammation, and tissue repair [46,47]. Their development, trafficking, and effector functions are tightly regulated by neuroendocrine signals. Chronic activation of the HPA axis and SAM system leads to sustained exposure to glucocorticoids and catecholamines, profoundly altering the functional state of both cell types.

##### Macrophages

Macrophages exhibit marked functional plasticity, enabling them to adopt a spectrum of activation states in response to environmental cues [48]. Cytokines produced by TH1 lymphocytes promote a classically activated (M1) phenotype with pro-inflammatory and antimicrobial properties, whereas IL-4 and IL-13 from TH2 lymphocytes favor an alternatively activated (M2) phenotype associated with anti-inflammatory functions. This M1/M2 classification reflects a simplified continuum of macrophage phenotypes rather than a rigid dichotomy [49,50]. In addition to classical inflammatory signaling pathways such as NF-κB, mitogen-activated protein kinase (MAPK), and JAK/STAT, chronic stress also affects macrophage metabolic programming through PI3K/Akt/mTOR and AMPK-dependent pathways [51,52]. Consequently, glucocorticoid-mediated inhibition of NF-κB signaling may become dysregulated, allowing persistent production of pro-inflammatory cytokines such as IL-1β, IL-6, and TNF-α [38]. Catecholamines further modulate macrophage activity via cyclic AMP-dependent signaling pathways, influencing cytokine production and inflammatory responses [45]. Chronic stress also induces metabolic reprogramming of macrophages toward increased glycolysis, a hallmark of pro-inflammatory activation that sustains cytokine production and reactive oxygen species (ROS) generation, contributing to tissue damage and impaired resolution of inflammation [52,53,54]. Functionally, chronic stress impairs innate immune function, including macrophage-mediated phagocytosis, antigen presentation, and pathogen clearance [39,41,42]. Reduced antigen-presenting capacity, including decreased expression of major histocompatibility complex class II (MHC II) molecules, limits effective activation of adaptive immune responses [39,41]. Moreover, prolonged stress alters macrophage behavior within tissue-specific niches, particularly in the bone marrow. Macrophages residing in erythroblastic islands are essential for erythroid progenitor survival and differentiation through direct cell–cell interactions and secretion of regulatory factors. Chronic psychological stress has been shown to disrupt this microenvironment through altered macrophage activity and extracellular ATP signaling, thereby impairing erythropoiesis and contributing to hematopoietic imbalance [55].

##### Neutrophils

Neutrophils are the most abundant circulating leukocytes and serve as rapid responders to infection and tissue injury. Chronic stress significantly alters neutrophil homeostasis, leading to both quantitative and qualitative changes. Sustained glucocorticoid exposure delays neutrophil apoptosis, resulting in increased circulating neutrophil counts (neutrophilia) [47]. However, despite their elevated numbers, neutrophil functional capacity is compromised. Glucocorticoids and catecholamines impair neutrophil chemotaxis by altering the expression of adhesion molecules and chemokine receptors, thereby reducing efficient migration to sites of inflammation [56,57]. Chronic stress further disrupts neutrophil effector functions, including phagocytosis, degranulation, and oxidative burst activity [41,47]. Reduced NADPH oxidase (NOX2)-mediated ROS generation consequently impairs microbial killing [47,58]. Chronic stress also promotes dysregulated formation of neutrophil extracellular traps (NETs), a process known as NETosis [42,59]. Although NETs play an important role in trapping and neutralizing pathogens, excessive NET formation contributes to endothelial dysfunction, vascular inflammation, and thrombosis [59]. This mechanism has been increasingly implicated in stress-related cardiovascular pathology [3,39,60].

##### Macrophage–Neutrophil Interactions Under Chronic Stress

Macrophages and neutrophils act in a coordinated manner during immune responses, regulating both the initiation and resolution of inflammation [61]. Chronic stress disrupts this crosstalk, contributing to immune dysregulation. Dysregulated macrophage activation promotes aberrant neutrophil recruitment and activation, while neutrophils modulate macrophage function through the release of ROS, proteases, and NET-associated components, amplifying pro-inflammatory signaling pathways [58,61]. A critical step in the resolution of inflammation is the clearance of apoptotic neutrophils (efferocytosis) by macrophages [55,61,62]. Chronic stress impairs this process, leading to the accumulation of dying cells, secondary tissue damage, and sustained inflammatory signaling [55]. Dysfunctional macrophage–neutrophil interactions link chronic stress to systemic disease. Persistent inflammation and impaired pathogen clearance contribute to cardiovascular, metabolic, neurodegenerative, and malignant disorders [3,4,5,6,12,14]. Cytokines produced by these immune cells can also activate the HPA axis, reinforcing neuroendocrine responses and establishing a maladaptive stress–immune feedback loop [7,8].

#### 2.2.3. Stress Erythropoiesis and Erythroid Progenitors: An Emerging Neuroimmune Hypothesis

Chronic stress may also affect hematopoietic and erythroid compartments. Sustained HPA/SAM activation, glucocorticoid signaling, inflammatory cytokines, and bone marrow niche alterations can activate stress erythropoiesis and promote expansion of erythroid progenitor populations [63,64,65,66,67]. In murine models of chronic psychological stress, extramedullary erythropoiesis and erythroid progenitor expansion have been linked to macrophage-associated mechanisms, nitric oxide-dependent pathways, and purinergic signaling [55,68,69]. Since immature CD71^+^ erythroid cells may exert immunosuppressive effects and impair immune surveillance in infection and cancer-related contexts [70,71,72,73,74], stress-induced erythroid progenitors may represent an emerging link between erythropoietic dysregulation and immune imbalance. However, direct evidence that terpenes modulate stress erythropoiesis or erythroid progenitor function is currently lacking. Therefore, this connection is presented as a hypothesis-generating extension of the neuroimmune framework rather than as an established mechanism of terpene action.

#### 2.2.4. Cytokines as Mediators of Stress-Induced Immune Dysregulation

Cytokines represent a central communication network between the immune system, the HPA axis, and the central nervous system, enabling bidirectional communication that is essential for maintaining physiological homeostasis during stress exposure. Under acute stress, cytokine signaling contributes to adaptive immune modulation and transient activation of inflammatory pathways [43]. However, chronic stress may disrupt cytokine balance and promote sustained immune activation and a pro-inflammatory milieu, depending on stressor duration, intensity, biological context, and disease state [39,43]. Chronic stress is associated with increased circulating and tissue levels of key pro-inflammatory cytokines, including interleukin-1β (IL-1β), interleukin-6 (IL-6), and TNF-α [75]. These pro-inflammatory cytokines can stimulate hypothalamic CRH synthesis and release, thereby activating the HPA axis and reinforcing a maladaptive feed-forward loop between inflammation and neuroendocrine stress responses. IL-6 has been shown to activate the HPA axis directly, sometimes more potently than CRH, indicating a robust cytokine-neuroendocrine interaction pathway in chronic inflammatory states [76].

Cytokines can influence the brain through several pathways. They may cross the blood–brain barrier via active transport mechanisms, access the brain at circumventricular organs where the barrier is naturally more permeable, or signal indirectly through vagal afferent pathways. Once within the central nervous system, cytokines can activate microglia, compromise blood–brain barrier integrity, and modulate neurotransmitter metabolism, thereby contributing to changes in neural function [77]. These processes impair synaptic plasticity, reduce neurogenesis, particularly in the hippocampus, and interfere with monoaminergic signaling pathways involved in mood regulation and cognition [78]. As a result, chronic stress-induced cytokine dysregulation is increasingly recognized as a key biological mechanism linking prolonged psychological stress to depression, cognitive decline, and neurodegenerative processes [77,79].

#### 2.2.5. T and B Lymphocytes in Chronic Stress-Induced Immune Suppression

Adaptive immunity is particularly vulnerable to chronic stress-induced neuroendocrine dysregulation. Prolonged activation of the HPA axis and SAM system disrupts lymphocyte development, trafficking, proliferation, and effector function, resulting in impaired antigen-specific immune responses [39,80]. Glucocorticoids suppress T-cell activity by inhibiting T-cell receptor (TCR) signaling, reducing cytokine transcription required for clonal expansion, and promoting apoptosis of immature and activated T-cells. Chronic stress decreases T-cell proliferation and shifts T helper cell differentiation from a Th1-dominant, cell-mediated immune response toward a Th2-skewed humoral profile. This shift is characterized by reduced production of TNF-α, IFN-γ, and IL-2, and relative preservation or enhancement of Th2-associated cytokines such as IL-4 and IL-10 [80]. This Th1/Th2 imbalance compromises cytotoxic T-lymphocyte activity, weakens antiviral and antitumor immunity, and may facilitate immune escape and disease progression in cancer and other stress-related disorders [80].

B lymphocytes are similarly affected. Glucocorticoids impair B-cell development in the bone marrow and antibody production by inhibiting class-switch recombination and plasma cell differentiation [81]. Chronic stress is associated with altered immunoglobulin profiles, reduced vaccine responsiveness, and impaired immunological memory [82]. It also disrupts lymphocyte trafficking by altering adhesion molecule and chemokine receptor expression, causing redistribution of T and B-cells away from peripheral tissues and secondary lymphoid organs, thereby limiting effective immune surveillance and adaptive coordination [56,83].

### 2.3. Stress Effects on Nervous Tissue

Beyond its systemic immunological effects, chronic stress can substantially affect the central nervous system (CNS), although the nature and magnitude of these effects may vary according to stressor type, age, sex, disease state, and study design. The CNS not only serves as a primary regulator of the stress response, but also represents one of its main targets, functioning in close and dynamic interaction with the immune system [8]. Although acute stress elicits adaptive neural changes that enhance survival, learning, and memory consolidation, chronic stress leads to sustained neuroendocrine activation that disrupts neuronal homeostasis and brain plasticity [84,85]. The brain is particularly vulnerable during sensitive developmental and aging periods, when stress exposure can exert long-lasting effects on neural structure and function [86]. Prolonged activation of the HPA axis and sympathetic nervous system exposes neural tissue to persistently elevated glucocorticoids and catecholamines, which alter excitatory and inhibitory neurotransmission, leading to dysregulation of glutamatergic, GABAergic signaling, and monoaminergic systems involved in stress responsivity [87,88,89]. Chronic stress also impairs synaptic integrity and neurotrophic support, disturbs cellular metabolism and mitochondrial function, and promotes neuroinflammatory processes through microglial activation [90,91,92,93]. Because stress-sensitive brain regions such as the hippocampus, prefrontal cortex, and amygdala are densely populated with glucocorticoid receptors, they are particularly vulnerable to chronic hormonal and inflammatory perturbations [9]. Over time, these molecular and cellular changes translate into structural remodeling, impaired neural connectivity, and functional deficits that underlie the cognitive, emotional, and behavioral consequences of chronic stress. Elucidating the neurotransmitter, molecular, and structural changes induced by prolonged HPA activation highlights pathways that terpenes may modulate to protect neural integrity and cognitive function. In other words, these pathways represent potential therapeutic targets for interventions aimed at restoring neuroendocrine and immune balance under chronic stress conditions.

#### 2.3.1. Neurotransmitter Alteration

Chronic or repeated stress exposure has been associated with alterations in neurotransmitter systems that regulate mood, cognition, motivation, and neuroendocrine function. Glucocorticoids exert complex control over monoaminergic systems, influencing monoamine synthesis, presynaptic release, reuptake, and postsynaptic receptor responsiveness. As a result, chronic stress is associated with reduced serotonergic transmission and altered receptor expression, which is strongly implicated in the pathophysiology of major depressive and anxiety disorders [90]. Dopaminergic signaling within the mesolimbic and mesocortical pathways is likewise disrupted, contributing to anhedonia and motivational deficits. In addition, chronic stress enhances noradrenergic tone through sustained activation of the locus coeruleus, the brain’s primary source of norepinephrine, promoting hypervigilance, anxiety, and disruptions in arousal and sleep regulation [8,88]. Beyond monoamines, chronic stress dysregulates excitatory and inhibitory neurotransmission. Excessive glucocorticoid exposure increases extracellular glutamate levels in stress-sensitive regions, partly by impairing astrocytic uptake and enhancing presynaptic release [87]. This excitotoxic environment promotes dendritic retraction and neuronal vulnerability through overactivation of NMDA receptors. Concurrently, GABAergic signaling may be reduced, weakening the brain’s capacity to buffer stress-induced excitation and further amplifying HPA axis activation [89]. These neurotransmitter alterations disrupt neural network stability and help to explain the cognitive and behavioral consequences of chronic stress.

#### 2.3.2. Cellular and Molecular Changes: Neuroinflammation

As outlined in the previous section, chronic stress may induce glucocorticoid resistance at the cellular level, diminishing the anti-inflammatory effects of cortisol despite sustained elevations in circulating hormone levels [40]. Chronic stress triggers cellular and molecular alterations within the central nervous system that promote neuroinflammation. Microglia, the resident immune cells of the brain, are highly sensitive to both stress hormones and peripheral inflammatory mediators. Prolonged stress promotes their activation toward predominantly pro-inflammatory states, characterized by increased production of IL-1β, IL-6, TNF-α, and reactive oxygen and nitrogen species [93,94], although microglial phenotypes exist along a dynamic and context-dependent spectrum.

Activated microglia influence neighboring astrocytes, inducing reactive phenotypes that amplify local inflammatory cascades and reduce support for neuronal function [95]. Stress also increases blood–brain barrier (BBB) permeability, allowing peripheral immune mediators and monocytes to enter the brain and further stimulate glial activation [96]. At the molecular level, sustained elevations of glucocorticoids and pro-inflammatory cytokines impair mitochondrial respiration, increase oxidative stress, and weaken antioxidant defenses [92,97]. Mitochondria act as glucocorticoid-sensitive signaling hubs, integrating metabolic and inflammatory cues that shape neuronal resilience or vulnerability. Persistent oxidative imbalance results in damage to lipids, proteins, and nucleic acids, promoting synaptic dysfunction and neuronal atrophy [98]. Chronic stress further activates transcription factors such as NF-κB, a crucial regulator of innate immunity, cell survival, and proliferation, thereby sustaining cytokine production [93]. In addition, inflammation-induced activation of indoleamine 2,3-dioxygenase (IDO) in brain immune cells diverts tryptophan metabolism from serotonin synthesis toward the kynurenine pathway. This shift reduces serotonin availability while increasing neuroactive kynurenine metabolites, some of which exert neurotoxic effects and exacerbate neuroinflammation, synaptic impairment, and disrupted neuronal plasticity [79]. Moreover, chronic stress reduces the expression of brain-derived neurotrophic factor (BDNF), a key mediator of neuronal survival and synaptic plasticity, further promoting structural and functional neural deterioration [91].

#### 2.3.3. Structural Changes in the Brain

Sustained activation of the HPA axis, together with neuroinflammatory processes, leads to measurable structural alterations in stress-sensitive brain regions [8,86]. The hippocampus is particularly vulnerable due to its high density of glucocorticoid and mineralocorticoid receptors [9,84]. Chronic stress has been associated with dendritic atrophy, reduced spine density, and suppression of adult hippocampal neurogenesis within the dentate gyrus [84,99]. Because the hippocampus plays a central role in negative feedback regulation of the HPA axis, these alterations may weaken inhibitory control over hypothalamic stress circuits, thereby perpetuating glucocorticoid dysregulation. Over time, such changes may culminate in reduced hippocampal volume, a finding consistently reported in major depressive disorder, post-traumatic stress disorder, and other chronic stress-related conditions [100,101]. The prefrontal cortex (PFC), which mediates executive functions such as planning, decision-making, impulse control, social cognition, and emotional regulation, is likewise highly sensitive to prolonged stress exposure. In this region, chronic stress induces dendritic retraction and synaptic weakening within medial PFC circuits, impairing top-down control over limbic and hypothalamic stress-responsive regions [102]. In contrast, the amygdala often exhibits dendritic hypertrophy and increased spine density under chronic stress, changes that enhance anxiety-like behavior [85,103]. These region-specific adaptations—reduced hippocampal and prefrontal control and amygdala hyperactivity—drive heightened stress responsivity and impaired neuroendocrine feedback, sustaining HPA axis dysregulation [8,85].

#### 2.3.4. Functional Effects and Clinical Consequences

The cumulative neuroinflammatory and structural alterations induced by chronic stress culminate in significant functional impairments. Disrupted hippocampal and prefrontal cortex function contributes to deficits in memory consolidation, attention, executive function, and decision-making. Heightened amygdala reactivity promotes anxiety, emotional lability, and exaggerated stress responses [85,100,103]. Chronic stress-induced neuroinflammation and neurotransmitter dysregulation are strongly implicated in the pathogenesis of major depressive disorder, anxiety disorders, post-traumatic stress disorder, and stress-related cognitive decline [90,91]. Furthermore, prolonged exposure to glucocorticoids may accelerate neuronal aging and increase vulnerability to neurodegenerative diseases such as Alzheimer’s and Parkinson’s disease. These effects are linked to enhanced amyloidogenesis, tau phosphorylation, mitochondrial dysfunction, and synaptic loss [13]. Collectively, chronic stress induces system-level dysregulation characterized by glucocorticoid receptor resistance, persistent NF-κB activation, mitochondrial dysfunction, impaired neurotrophic signaling, and maladaptive structural remodeling within stress-sensitive brain regions. These interconnected processes form a self-reinforcing neuroendocrine–immune network that sustains HPA axis hyperactivity and chronic inflammation. Such convergence identifies shared molecular nodes, including GR signaling, NF-κB-dependent transcription, IDO-mediated tryptophan metabolism, oxidative stress pathways, and BDNF regulation, as biologically coherent therapeutic targets. A comprehensive overview of chronic stress-induced dysregulation across endocrine, immune, metabolic, hematopoietic, and nervous systems is presented in Table 1.

## 3. Terpenes: General Characteristics and Biological Relevance

Terpenes represent a large and structurally diverse class of naturally occurring organic compounds primarily synthesized by plants as secondary metabolites [16]. They are derived from isoprene units and are classified according to their carbon structure into hemiterpenes, monoterpenes, sesquiterpenes, diterpenes, and triterpenes [16,104]. Terpenoids are modified terpenes, derived from isoprene units and characterized by the presence of additional functional groups, typically oxygen-containing groups [105]. Many terpenes and terpenoids are lipophilic, allowing them to cross biological membranes, including the blood–brain barrier, and exert direct effects within the central nervous system [22,106]. They constitute the major fraction of essential oils and are responsible for the characteristic aroma and flavor of many plant species. In plants, they also contribute to defense mechanisms, pollinator attraction, and interspecies communication [107,108]. In mammalian experimental models, isolated terpenes, selected terpenoid compounds, and terpene-rich preparations have been reported to exert biological activities relevant to neural, immune, and stress-related regulatory pathways. Beyond these ecological and physiological functions, terpenes and terpenoids interact with multiple molecular targets, including transcription factors, cytokine networks, and redox-sensitive signaling pathways [18,19,104]. Preclinical and selected human studies suggest that terpene-related exposures may influence pathways relevant to stress-related neuroimmune and redox regulation; however, direct evidence that isolated terpenes modulate endocrine outcomes in humans remains limited. [21,22]. An overview of the major terpene representatives and their multi-system effects is provided in Table 2.

The following sections summarize their effects on immune, nervous, and endocrine systems, with emphasis on stress-related pathways.

### 3.1. Terpenes and the Immune System

Terpenes and terpenoids are plant-derived compounds with reported immunomodulatory and anti-inflammatory properties, mainly documented in in vitro and preclinical models. Depending on the compound, preparation, and experimental system, they may influence immune responses by modulating cytokine production, intracellular signaling, redox balance, and inflammatory gene expression [112,121].

#### 3.1.1. Effects of Terpenes and Terpenoids on Anti-Inflammatory Signaling Pathways

A central mechanism underlying the immunomodulatory activity of terpenes is the regulation of intracellular inflammatory signaling pathways. Monoterpenes commonly inhibit NF-κB and MAPK pathways, which are key drivers of pro-inflammatory cytokine production, including TNF-α, IL-1β, and IL-6 [18,19,121]. Dysregulated NF-κB signaling is a hallmark of chronic stress-induced inflammation and is closely linked to impaired glucocorticoid receptor function [38,40,75]. Linalool, one of the most extensively studied monoterpenoids, reduces inflammation in multiple experimental models, including carrageenan-induced edema and LPS-induced tissue injury, primarily through inhibition of NF-κB activation and downregulation of inducible nitric oxide synthase (iNOS) and cyclooxygenase-2 (COX-2) [116,117]. It also suppresses inflammatory responses in LPS-stimulated macrophages by decreasing TNF-α and IL-6 production and inhibiting MAPK phosphorylation [118]. In addition, linalool activates the nuclear factor erythroid 2-related factor 2 (Nrf2) pathway, thereby enhancing antioxidant defenses and linking anti-inflammatory and redox-regulatory mechanisms [117]. Monoterpenes such as α-pinene, limonene, myrcene, and terpenoid 1,8-cineole exhibit similar anti-inflammatory properties [112]. These compounds inhibit NF-κB activation and related signaling cascades, resulting in reduced expression of pro-inflammatory mediators [122,123,124,125]. Notably, 1,8-cineole has demonstrated clinical relevance by reducing glucocorticoid requirements and improving pulmonary function in asthma patients [115]. Through suppression of pro-inflammatory cytokines, terpenes may indirectly modulate HPA axis activation. In addition, regulation of NF-κB signaling may theoretically contribute to improved glucocorticoid responsiveness in immune cells; however, direct evidence demonstrating that terpenes can restore glucocorticoid sensitivity in chronic stress models is currently lacking.

#### 3.1.2. Effects of Terpenes on Innate Immune Cells

The modulation of inflammatory signaling pathways by terpenes translates into functional effects on innate immune cells, particularly under chronic stress.

##### Macrophages

Macrophages are highly plastic regulators of innate immunity and tissue homeostasis. Chronic stress disrupts macrophage function, promoting pro-inflammatory metabolic states and impairing resolution of inflammation [42,53,54]. Terpenoids modulate macrophage polarization and inflammatory signaling. Linalool suppresses pro-inflammatory cytokine production and inhibits NF-κB and MAPK pathways in activated macrophages [112,118]. Lupeol promotes a shift from pro-inflammatory M1 to anti-inflammatory M2 phenotypes, reducing inflammatory mediators and enhancing regulatory markers [126]. By modulating pro-inflammatory and metabolic signals, as well as redox status, terpenes may promote a more balanced macrophage phenotype that limits chronic inflammation. Whether these effects influence erythroid progenitor maintenance in specialized bone marrow niches remains unknown and requires direct experimental investigation.

##### Neutrophils

Neutrophils are also affected by terpene-mediated modulation and are susceptible to stress-induced dysfunction, including impaired chemotaxis and reduced microbial killing capacity [47]. Although direct studies on terpene effects on neutrophils remain relatively limited, several investigations suggest that terpenoids can regulate neutrophil-mediated inflammatory responses. Terpene-rich essential oils have been shown to suppress TNF-α-mediated neutrophil adherence, thereby limiting excessive activation and subsequent tissue damage [127]. Borneol has been reported to modulate neutrophil signaling through calcium flux and FPR1/FPR2 pathways, resulting in reduced chemotaxis and functional desensitization [128]. Terpenoids may also reduce reactive oxygen species production and limit neutrophil extracellular trap formation, thereby attenuating inflammatory amplification [59,112].

##### NK Cells

Natural killer (NK) cells are essential for antiviral and antitumor immunity but are suppressed under chronic stress [44,45]. Several studies have demonstrated that phytoncides, complex mixtures of volatile compounds, including terpenes and terpenoids, emitted by forest trees, can enhance NK cell activity [129,130]. Exposure of human NK cell lines to phytoncides increased cytolytic activity by upregulating expression of perforin, granzyme A, and granulysin [129]. Furthermore, α-pinene activates ERK and Akt signaling pathways in NK-92MI cells, upregulating activation markers such as CD56 and CD107a and enhancing NK cell cytotoxic function [130]. These findings suggest that selected terpene-related exposures may enhance NK-cell activation and cytotoxicity in experimental systems. However, evidence demonstrating reversal of stress-associated NK-cell suppression in chronic stress models remains limited.

#### 3.1.3. Effects of Terpenes on Adaptive Immunity

Terpenes also influence adaptive immune responses. Chronic stress alters adaptive immunity by suppressing T cell-mediated responses and promoting a Th2-dominant profile, marked by increased IL-4, IL-5, and IL-10 production and reduced expression of Th1-associated cytokines such as IFN-γ and IL-2 [80]. This imbalance weakens cell-mediated immunity and antibody production [81,82]. Terpenes influence adaptive immune responses by modulating cytokine secretion profiles. In vitro studies demonstrate that multiple terpenoid compounds modulate Th1/Th2 cytokine balances, reducing pro-inflammatory bias and supporting more regulated immune responses [131]. Certain terpenes demonstrate additional immunomodulatory mechanisms through interaction with specific immune receptors [113]. β-caryophyllene, for example, acts as a selective agonist of the cannabinoid CB2 receptor, which is predominantly expressed on immune cells [113]. Through this mechanism, β-caryophyllene may exert anti-inflammatory and immunoregulatory effects that can modulate both T- and B-cell activity [113].

#### 3.1.4. Antioxidative Activity of Terpenes

Oxidative stress is a key mechanism linking chronic stress, immune dysregulation, and tissue damage. Prolonged activation of the HPA axis increases reactive oxygen species (ROS) production, contributing to cellular damage and inflammation [92,97,98]. Terpenes exhibit significant antioxidant activity, acting both as direct free radical scavengers and as modulators of endogenous antioxidant defenses [104]. Phenolic and oxygenated terpenes, such as thymol, carvacrol, and carnosic acid, demonstrate particularly strong antioxidant capacity, largely due to structural features such as hydroxyl groups and conjugated double bonds [104]. Linalool, limonene, and α-pinene have been shown to enhance endogenous antioxidant defense systems by increasing the activity of key antioxidant enzymes such as superoxide dismutase (SOD), catalase, and glutathione peroxidase [17,104]. Linalool treatment significantly enhanced catalase activity in an experimental model of diabetic rats [132]. Many studies also revealed that D-limonene provides antioxidant properties, inhibits lipid peroxidation, and fights against free radical-induced cell damage [109,110,133,134]. α-pinene has been shown to restore antioxidant enzyme activity and reduce inflammation in an animal model of ischemia [135]. 1,8-cineole and α-pinene also protect against H_2_O_2_-induced oxidative stress in PC12 cell line [136]. Terpenes further modulate redox-sensitive signaling pathways, particularly the Nrf2 and heme-oxygenase 1 (HO-1) pathway, contributing to the restoration of redox homeostasis [136,137]. Beyond their antioxidant and anti-inflammatory properties, emerging evidence suggests that certain monoterpenes may also influence erythroid differentiation. Selected monoterpenes (carvacrol, 3-carene and 1,4-cineole) have been reported to promote erythroid maturation and enhance γ-globin gene expression and fetal hemoglobin synthesis [138]. Although selected monoterpenes have been reported to influence erythroid maturation and fetal hemoglobin synthesis in experimental systems [138], it remains unknown whether terpene-related interventions modulate stress erythropoiesis or immunomodulatory erythroid progenitor function under chronic stress. This potential connection should therefore be considered hypothesis-generating and requires targeted experimental validation.

### 3.2. Terpenes and the Nervous System

Isolated terpenes, selected terpenoid compounds, and terpene-rich preparations have shown neurobiological effects in experimental and selected human studies that may be relevant to prolonged stress exposure and HPA axis dysregulation. Once in the central nervous system, these compounds can directly interact with neuronal and glial cells, modulating neurotransmission and neuroinflammatory signaling, including pathways associated with the endocannabinoid system, a key regulator of stress and neural homeostasis [139].

#### 3.2.1. Neurotransmitter Modulation

Terpenes and terpenoids influence CNS function through modulation of neurotransmitter systems involved in stress responses, including GABAergic, glutamatergic, and monoaminergic signaling. Linalool has been reported to attenuate glutamate- and NMDA-mediated neurotoxicity and excitotoxic neuronal damage in experimental models, thereby contributing to neuroprotection under conditions associated with excessive excitatory signaling [140]. It also enhances GABAergic transmission, increasing inhibitory tone and producing anxiolytic effects [119]. Limonene has been reported to modulate neurotransmitter levels and behavior in rats. Specifically, it was demonstrated that s-limonene suppresses HPA axis activity under conditions of physical stress, with evidence suggesting that its anti-stress effects are mediated via the GABAA receptor [141]. α-Pinene modulates dopaminergic transmission and has been linked to improved cognitive performance [142]. These effects suggest potential relevance in mitigating stress-induced anhedonia and cognitive impairment. Additionally, some terpenes also influence cholinergic signaling. 1,8-cineole exhibits mild acetylcholinesterase inhibition, likely enhanced through synergistic interactions in essential oils [143]. Myrtenal shows stronger inhibitory activity, suggesting that multiple components act together to enhance cholinergic neurotransmission [144].

#### 3.2.2. Neuroprotective and Antioxidant Effects

In preclinical CNS and neurodegenerative disease models, several isolated terpenes and selected terpenoid compounds have been reported to exert neuroprotective effects partly through modulation of redox homeostasis, neuroinflammatory signaling, and neuronal survival pathways. They enhance endogenous antioxidant defenses and reduce ROS accumulation, thereby protecting neuronal structures from oxidative damage [104,106]. β-caryophyllene has been shown to attenuate oxidative stress and neuroinflammation via CB2 receptor-mediated mechanisms [145,146]. Terpenes exert anti-inflammatory actions by suppressing microglial activation and pro-inflammatory cytokine production [19,147], which may be relevant in stress-related cognitive decline. Neuroprotective effects have been demonstrated in neurodegenerative disease models. Linalool reduces amyloid-β accumulation, glial activation, and neuronal loss while improving cognitive function in Alzheimer’s disease models [148,149,150]. Similarly, α-pinene and limonene show similar protective effects by reducing oxidative stress and improving neuronal survival [151,152].

#### 3.2.3. Anxiolytic and Sedative Effects

In preclinical studies, selected terpenes and, in some cases, terpene-rich preparations have been reported to produce anxiolytic-like, antidepressant-like, and sedative or central depressant effects. These effects are relevant to stress-related anxiety and sleep disturbances, where imbalance between excitatory and inhibitory neural signaling contributes to hyperarousal. Linalool has been shown to produce anxiolytic-like effects in animal models, with minimal impairment of motor coordination at certain doses [119,120]. Limonene and β-caryophyllene also demonstrate anxiolytic and antidepressant-like effects in preclinical studies, the latter via CB2 receptor activation, supporting a role of the endocannabinoid system in emotional regulation and stress adaptation [111,113,114]. Sedative and central depressant effects have been reported for myrcene, citral, limonene, and linalool in animal models, including reductions in locomotor activity and anxiolytic-like behaviors [120,153]. However, at higher doses these compounds may induce increased sedation and reduced motor activity [153]. Overall, terpene effects arise from multi-target interactions involving neurotransmitter systems, endocannabinoid signaling, and neuroendocrine pathways. While these findings support their potential as adjunctive agents in managing stress-related neuropsychiatric symptoms, further clinical studies are required to establish their efficacy and safety in humans.

### 3.3. Terpenes and Indirect Neuroendocrine Regulation Under Chronic Stress

Under chronic stress conditions, terpene-related effects on neural and immune signaling may influence endocrine regulation primarily through central neurobiological pathways rather than direct hormonal interactions. Terpene-rich essential oils and selected terpene constituents may influence stress physiology through olfactory–limbic and autonomic pathways, leading to downstream modulation of neuroinflammatory and redox balance, as well as mood and sleep-related outcomes. Because endocrine homeostasis is highly sensitive to inflammatory and neural inputs, such effects may, in turn, modify HPA axis activity and related endocrine systems. However, evidence that isolated terpenes consistently normalize circulating endocrine hormones under chronic stress remains limited, including key components of the HPA axis (ACTH, cortisol), adrenal steroids (DHEA/DHEA-S), gonadal and thyroid hormones, prolactin, growth hormone, and metabolic hormones (insulin, leptin, ghrelin), as well as catecholamines [19,20,154].

The strongest endocrine-related evidence concerns HPA axis-associated stress responses. Human and experimental studies of terpene-rich essential oils, particularly lavender, citrus, rosemary and bergamot preparations, report reductions in perceived stress and anxiety-like behaviors, and in some cases changes in cortisol-related markers [20,155]. These effects are biologically plausible, as volatile terpenes can engage olfactory pathways linked to limbic and hypothalamic regions involved in emotional and stress regulation. However, most available data derive from complex essential oil mixtures rather than isolated terpene compounds, while endocrine outcomes are often secondary, heterogeneous, or inconsistently reported. Therefore, current evidence supports terpene-rich essential oil exposure as a potential modulator of stress responsivity and selected HPA axis-related outcomes [20,155], whereas evidence that isolated terpene compounds directly regulate cortisol secretion remains insufficient [20,154,155].

Metabolic endocrine regulation represents another relevant domain of terpene action under chronic stress. Chronic stress may promote metabolic dysfunction through prolonged glucocorticoid exposure, low-grade inflammation, oxidative stress, altered appetite-related signaling, insulin resistance, and visceral adiposity [26]. Some terpenes, particularly β-caryophyllene, have shown anti-inflammatory and metabolic effects in preclinical models, partly through CB2 receptor activation and PPARγ-related pathways [156,157]. These effects may be relevant to stress-associated metabolic dysregulation by attenuating inflammatory signaling, improving redox balance, and supporting immune–metabolic homeostasis. However, evidence for direct regulation of insulin, leptin, ghrelin, or other metabolic hormones in chronically stressed humans remains insufficient.

**Figure 3:**
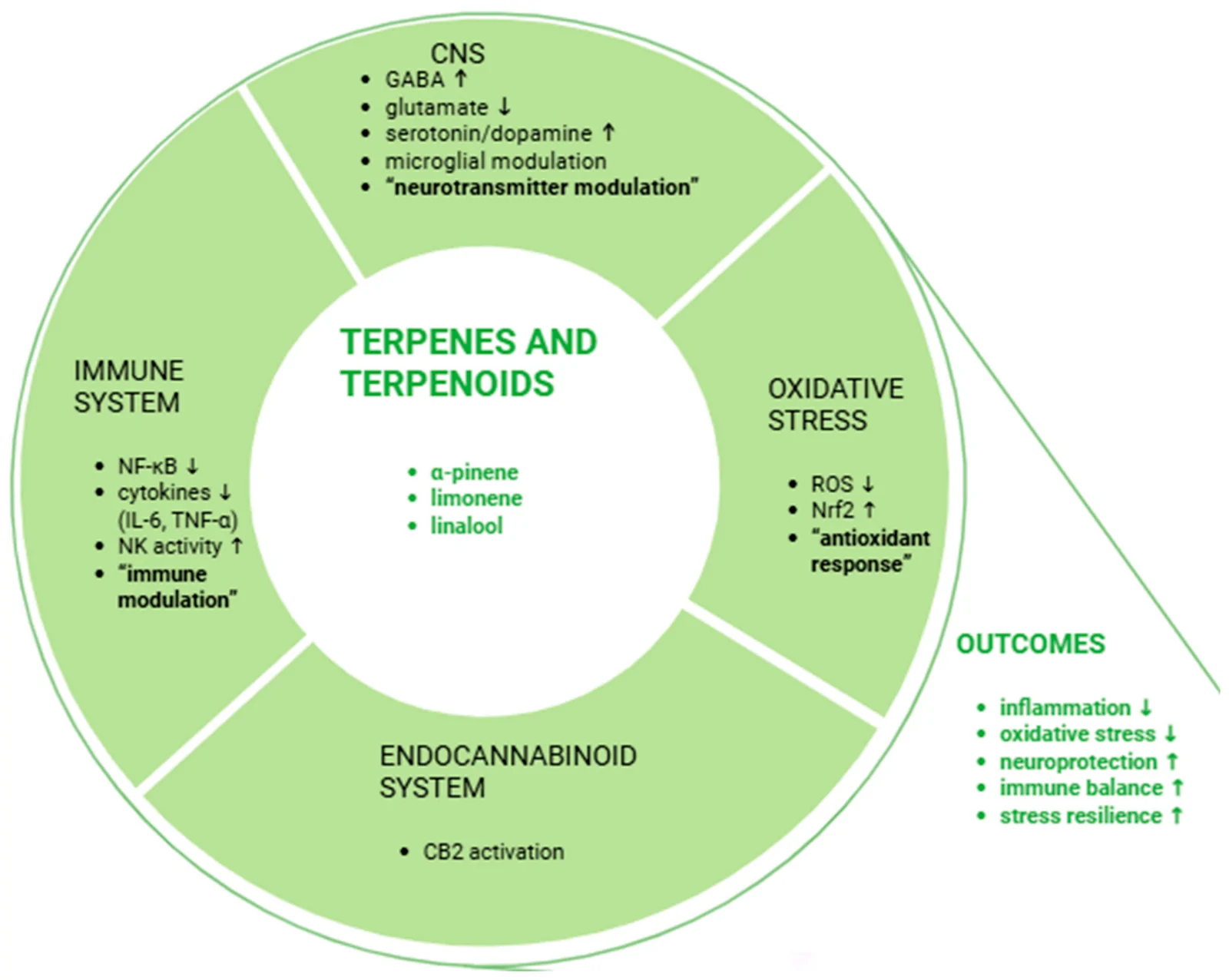
Multi-target effects of terpenes and terpenoids on stress-related pathways. The figure summarizes key pathways potentially modulated by terpenes and terpenoids in the context of chronic stress, including immune and inflammatory signaling (NF-κB, pro-inflammatory cytokines, NK-cell activity); oxidative stress and antioxidant responses (ROS, Nrf2); central nervous system (GABAergic, glutamatergic, serotonergic, and dopaminergic signaling) and microglial activity; and endocannabinoid-related pathways, including CB2-associated signaling. These interconnected mechanisms may contribute to outcomes relevant to stress-related disorders, including reduced neuroinflammation and oxidative stress, improved immune balance, enhanced neuroprotection, and increased stress resilience.

Evidence for terpene effects on reproductive hormones, DHEA/DHEA-S, thyroid hormones, prolactin, GH, or GH/IGF-1 signaling during chronic stress is also limited [158]. While anxiolytic, anti-inflammatory, antioxidant, and sleep-modulating properties of terpenes may indirectly influence these endocrine systems, current data do not support firm conclusions that isolated terpenes directly normalize HPG, HPT, adrenal androgen, or pituitary hormone function in chronically stressed humans [19,20,155]. Overall, terpenes are best viewed as multi-target modulators of stress-related neuroendocrine and immune pathways, with effects likely mediated through upstream regulatory mechanisms rather than directly regulating endocrine hormone secretion. These interactions are summarized in Figure 3.

### 3.4. Human Evidence for Terpene-Related Modulation of Stress-Related Outcomes

Compared with preclinical evidence, human data on terpene-related modulation of chronic stress pathways remain more limited and heterogeneous. The strongest available human evidence is derived mainly from studies of terpene-rich essential oils and forest-derived volatile exposure rather than from controlled trials using isolated terpene compounds. Human studies of lavender, citrus, rosemary, and bergamot essential oil preparations have reported improvements in perceived stress, anxiety-related symptoms, mood, sleep-related outcomes, autonomic stress markers, and, in some cases, cortisol-related measures [20,155]. These findings support the potential relevance of olfactory, limbic, and autonomic pathways in human stress regulation. Additional human evidence comes from forest-bathing and phytoncide-exposure studies, which have been associated with improved psychological well-being and modulation of selected immune parameters, including NK cell-related outcomes [25,129]. However, these studies represent complex environmental interventions that include physical activity, visual and sensory stimulation, relaxation, and contextual factors, in addition to inhalation of forest-derived volatile compounds. Therefore, they should not be interpreted as direct evidence for isolated terpene effects.

Overall, the strongest human evidence supports terpene-related exposures as potential modulators of perceived stress, affective symptoms, autonomic regulation, selected cortisol-related markers, and immune-related outcomes. However, direct clinical evidence that isolated terpenes modulate HPA axis hormones, inflammatory cytokines, or neuroendocrine–immune dysregulation in chronic stress remains insufficient. Thus, human findings are best interpreted as translationally relevant but indirect evidence, complementary to mechanistic preclinical data.

## 4. Translational and Safety Considerations

The translational relevance of terpene-related interventions depends on dose, route of administration, chemical standardization, metabolism, bioavailability, blood–brain barrier penetration, and safety. Purified terpene compounds, supplements containing terpenes, essential oils, and forest-derived volatile exposure should not be considered pharmacologically equivalent. Isolated compounds allow more precise evaluation of dose–response relationships, pharmacokinetics, metabolism, and target engagement, compared to complex essential oil mixtures whose composition may vary according to plant species, chemotype, extraction method, storage conditions, and variability [16,159,160]. Similarly, forest-derived phytoncide exposure represents a complex environmental intervention and cannot be directly equated with isolated terpene administration.

Route of administration is also critical. Inhaled volatile terpenes may engage olfactory, limbic, and autonomic pathways, while oral administration is influenced by gastrointestinal absorption, systemic bioavailability, and potential microbiome-dependent metabolism. Topical use introduces additional concerns related to skin absorption, irritation, and sensitization. Although many terpenes are lipophilic and may cross biological membranes, including the blood–brain barrier, data on pharmacokinetics, active metabolites, tissue distribution, central nervous system exposure, and elimination remain incomplete [22,106,159].

Safety requires careful consideration. Terpenes and essential oils are often perceived as natural and safe, but adverse effects may occur depending on dose, formulation, route, and individual susceptibility. Potential concerns include toxicity at high or repeated exposure levels, allergic or sensitization reactions, respiratory or gastrointestinal intolerance, excessive sedation with CNS-active compounds, and possible drug interactions, including effects of some terpene constituents on drug-metabolizing enzymes [160,161,162]. Therefore, future studies should use chemically standardized preparations, clearly define dose and route of administration, distinguish isolated compounds from supplements and essential oils, and systematically evaluate pharmacokinetics, bioavailability, blood–brain barrier penetration, toxicity, allergic reactions, and drug interaction potential.

At present, terpene-related interventions should not be regarded as replacements for established treatments for chronic stress-related disorders, including psychotherapy, antidepressant or anxiolytic pharmacotherapy, structured lifestyle interventions, and treatment of underlying medical conditions. Their most realistic translational role may be as adjunctive or supportive approaches, for example, through standardized essential oil inhalation protocols, carefully defined aromatherapy interventions, or structured forest-exposure programs. Such approaches may be relevant for perceived stress, sleep-related outcomes, affective symptoms, autonomic regulation, inflammatory signaling, oxidative stress, and neuroimmune balance. However, their integration with conventional therapies requires caution, particularly because of possible sedation, allergic or respiratory reactions, dose-dependent toxicity, and drug-interaction potential. Therefore, clinical implementation will require controlled human studies using chemically standardized preparations or isolated terpene compounds, clinically justified doses, pharmacokinetic and bioavailability data, safety monitoring, and validated clinical and mechanistic biomarkers. By identifying shared neuroimmune, redox, autonomic, and stress-responsive pathways, terpene-related research may help guide the development of future multi-target and biomarker-informed adjunctive strategies for stress-related disorders.

## 5. Limitations and Future Perspectives

Despite increasing interest in terpenes as modulators of stress-related neuroendocrine and immune dysfunction, several limitations remain in current research. Most available evidence derives from in vitro and animal studies, as well as investigations using complex essential oil preparations, although studies on isolated terpene compounds are also present. Many studies focus primarily on behavioral or inflammatory endpoints, whereas direct endocrine measures such as ACTH, cortisol dynamics, DHEA/DHEA-S balance, gonadal hormones, thyroid hormones, or autonomic biomarkers are often inconsistently assessed. The pharmacokinetic properties and bioavailability of many terpenes remain insufficiently characterized. Although many terpenes are lipophilic and capable of crossing the blood–brain barrier, their pharmacokinetics, bioavailability, and central nervous system distribution remain incompletely understood. Moreover, differences in administration route, terpene composition, and host-related factors such as sex, age, circadian timing, metabolic status, microbiome composition, and inflammatory conditions may substantially influence biological responses under chronic stress. Future research should prioritize standardized experimental designs and well-characterized terpene preparations to improve reproducibility across preclinical and clinical studies. Additional well-controlled clinical investigations are needed to clarify the relevance of anti-inflammatory, antioxidant, and neuroprotective effects observed in experimental models. Particular emphasis should be placed on endocrine and neuroimmune biomarkers, as well as on pharmacokinetic profiling and central nervous system distribution of individual terpenes.

## 6. Conclusions

Chronic stress may contribute to dysregulation of neuroendocrine, immune, metabolic, and neural pathways, with persistent HPA/SAM activation representing one important mechanism linking psychological stress exposure to systemic disease. Sustained glucocorticoid and catecholamine signaling contributes to glucocorticoid receptor resistance, chronic low-grade inflammation, oxidative stress, neuroinflammation, impaired immune surveillance, and maladaptive remodeling within stress-sensitive brain regions. These interconnected processes provide a biologically coherent framework through which chronic stress may increase vulnerability to neuropsychiatric, metabolic, cardiovascular, inflammatory, and neurodegenerative disorders. Current evidence indicates that terpenes and terpenoids interact with multiple components of this stress-responsive network. Through modulation of NF-κB-dependent inflammatory signaling, activation of antioxidant pathways such as Nrf2, regulation of microglial activity, modulation of neurotransmitter systems, and effects on immune-cell function, terpenes may attenuate several downstream consequences of chronic HPA axis dysregulation. In addition, certain terpenes influence stress-related behavioral responses, autonomic nervous system regulation, and neuroimmune communication, supporting their potential relevance as complementary modulators of stress-associated pathophysiology. However, current evidence remains predominantly preclinical, and direct endocrine effects of isolated terpenes on ACTH, cortisol, DHEA/DHEA-S, gonadal hormones, thyroid hormones, metabolic hormones, and catecholamines are not yet well established. Accordingly, terpenes and terpenoids should be interpreted primarily as potential indirect neuroimmune and redox modulators that may influence stress-related endocrine pathways through upstream inflammatory, oxidative, autonomic, and neuroimmune mechanisms, rather than as direct hormone-regulating agents. Further translational and clinical studies are required to define their pharmacological relevance, mechanistic specificity, and therapeutic applicability in stress-related disorders.

## Figures and Tables

**Table 1:** Overview of major systemic pathways affected by chronic stress, including endocrine, immune, metabolic, hematopoietic, and central nervous system alterations, along with key mediators and functional outcomes.

System	Key Dysregulated Pathways	Main Mediators	Functional Outcomes	References
Endocrine (HPA/HPG/HPT axis)	HPA axis hyperactivation, glucocorticoid resistance	Cortisol, ACTH, CRH, cytokines	Hormonal imbalance, metabolic dysregulation	[26,27,28,33,34]
Innate Immunity	NF-κB activation, glucocorticoid resistance	IL-6, TNF-α, IL-1β, catecholamines, NETs	Chronic low-grade inflammation, impaired immune response	[41,42,47,51,52,59]
Adaptive Immunity	Th1 → Th2 shift, lymphocyte suppression	Glucocorticoids, cytokines	Reduced antiviral and antitumor immunity	[56,80,83]
Central nervous system	Neuroinflammation, neurotransmitter dysregulation, blood–brain barrier (BBB) disruption, microglial activation	Cytokines, ROS, BDNF, monoamines (serotonin, dopamine, glutamate)	Synaptic dysfunction, cognitive impairment, anxiety, depressive symptoms	[89,90,91,92,93,94,97]
Metabolic Regulation	Insulin resistance, altered glucose/lipid metabolism, mitochondrial dysfunction, appetite dysregulation	Cortisol, catecholamines, leptin, insulin, inflammatory cytokines	Visceral adiposity, impaired energy homeostasis, metabolic syndrome risk	[26,28,92,97]
Hematopoietic System	Stress erythropoiesis, impaired erythroid differentiation	Glucocorticoids, inflammatory cytokines	Expansion of immature erythroid expansion, ineffective erythropoiesis, immunosuppression	[55,63,64,66,68,69]

**Table 2:** Summary of key terpenes and terpenoids, their primary system targets, and major biological effects.

	Affected Systems	Modulated Pathways	Functional Outcomes	References
Terpenes				
Limonene	CNS	Serotonergic Signaling	Anxiolytic, mood-modulating	[109,110,111]
α-Pinene	CNS, immune	ERK/Akt signaling, neuroimmune modulation	Neuroprotective, anti-inflammatory	[17,112]
β-Caryophyllene	Immune	CB2 receptor activation	Immunomodulatory, anti-inflammatory	[113,114]
Terpenoids				
1,8-Cineole	Immune, CNS	NF-κB inhibition, AChE inhibition	Anti-inflammatory, cholinergic modulation	[115]
Linalool	CNS, immune	GABA-A receptor modulation, NF-κB inhibition	Anxiolytic, anti-inflammatory	[116,117,118,119,120]

## Data Availability

No new data were created or analyzed in this study. Data sharing is not applicable to this article.

## Supplementary Materials

The following supporting information can be downloaded at: https://www.mdpi.com/article/10.3390/ijms27167242/s1.
