# Supplementary material for: Terpenes as Potential Multi-Target Modulators of Chronic Stress-Induced Neuroendocrine–Immune Dysregulation
Source: Int J Mol Sci. 2026 Aug 14;27(16):7242. doi: 10.3390/ijms27167242 (PMC13513126; doi:10.3390/ijms27167242)
Supplement: Supplementary file 1 [file ijms-27-07242-s001.zip › ijms-4402389-supplementary.pdf]

Table S1. **Classification of evidence categories**

(Supplementary Table S1)

**Compound/Preparation**	**Model/Population**	**Principal Outcomes**	**Relevance to Chronic Stress**	**Limitations**	**Evidence Category**	**Reference**
Linalool	Animal model: Experimental inflammation model	Anti-inflammatory activity	Relevant to stress-associated inflammation	Not a stress model	Preclinical Animal Evidence	[111]
Linalool	In vitro + animal model: RAW264.7 macrophages; LPS lung injury	Reduced TNF-α, IL-6, NF-κB and MAPK activity	Relevant to immune dysregulation	Acute inflammatory model	Preclinical Animal + In Vitro Evidence	[113]
Linalool	In vitro: BV2 microglia	Nrf2 activation; anti-inflammatory effects	Relevant to neuroinflammation	Cell model only	Mechanistic In Vitro Evidence	[133]
Linalool	In vitro/ex vivo: HT-22 neurons and hippocampal slices	Protection against glutamate- and NMDA-mediated toxicity	Relevant to stress-associated excitotoxicity	Not a chronic stress model	Mechanistic In Vitro/Ex Vivo Evidence	[136]
Linalool	Animal model (inhalation): Mice	Anxiolytic-like effects	Behavioral stress relevance	Animal model	Preclinical Animal Evidence	[137]
Linalool	Animal model (inhalation): Mice	Reduced anxiety-related behavior	Behavioral stress relevance	Animal model	Preclinical Animal Evidence	[150]
Limonene	Animal model (oral): Murine inflammation model	NF-κB inhibition; anti-inflammatory effects	Relevant to stress-related inflammation	Acute injury model	Preclinical Animal Evidence	[116]
S-limonene	Animal model (oral): Rats	Neurotransmitter modulation and anti-stress-like responses	Stress relevance	Animal model	Preclinical Animal Evidence	[138]
Limonene	Animal model + in vitro model: Mice; PC-12 cells	Anxiolytic activity via adenosine A2A receptor-mediated regulation	Stress relevance	Animal and cell-culture models	Preclinical Animal + In Vitro Evidence	[151]

Compound/Preparation	Model/Population	Principal Outcomes	Relevance to Chronic Stress	Limitations	Evidence Category	Reference
α-Pinene	In vitro: Murine macrophages	NF-κB/MAPK suppression	Relevant to chronic inflammation	Not a stress model	Mechanistic In Vitro Evidence	[115]
α-Pinene	Animal model + in vitro: Mice; NK-92MI cells	Enhanced NK-cell activation and cytotoxicity	Potential relevance to stress-related immune dysfunction	Animal and cell-culture model	Preclinical Animal + In Vitro Evidence	[123]
α-Pinene	Animal model: Rats with focal ischemic stroke	Reduced oxidative stress and inflammation	Relevant to stress-associated oxidative stress and neuroinflammation	Disease model	Preclinical Animal Evidence	[131]
β-Caryophyllene	Animal model + in vitro: Mice; Human monocytes	Selective CB2 agonist	Mechanistic relevance to stress-associated immune dysregulation and inflammation	Not a chronic stress model	Mechanistic Evidence	[125]
β-Caryophyllene	Animal model: Mice	Anxiolytic- and antidepressant-like effects	Stress relevance	Animal model	Preclinical Animal Evidence	[152]
β-Caryophyllene	Animal disease model: Rats	Reduced neuroinflammation and oxidative stress	Relevant to stress-associated neuroinflammation and oxidative stress	Disease model	Preclinical Animal Evidence	[142]
1,8-Cineole	Human clinical study: Asthma patients	Reduced glucocorticoid requirement; improved pulmonary function	Indirect relevance to stress-associated inflammation	Not a stress population	Primary Human Evidence (Clinical Study)	[118]
Phytoncides	In vitro: NK-92MI human cells	Increased NK-cell activity	Potential relevance to stress-associated immune dysfunction	Cell-culture model; no in vivo validation	Mechanistic In Vitro Evidence	[122]
Forest bathing	Narrative review: Human observational/interventional study: (healthy adults)	Improved well-being and selected immune outcomes	Translational relevance	Indirect evidence; multifactorial exposure	Review-Level Human Evidence	[25]

Compound/Preparation	Model/Population	Principal Outcomes	Relevance to Chronic Stress	Limitations	Evidence Category	Reference
Essential oils	Narrative review: Human and preclinical studies	Improved anxiety, mood, stress and sleep outcomes	Translational relevance	Heterogeneous evidence; review article	Review-Level Human Evidence	[20]
Plant-derived preparations	Systematic review: Human trials	Potential HPA-axis-related modulation	Direct relevance to HPA-axis dysregulation during chronic stress	Heterogeneous evidence	Review-Level Human Evidence	[21]
Essential oils	Scoping review: Human studies	Improved autonomic and stress-related outcomes	Translational relevance	Heterogeneous evidence	Review-Level Human Evidence	[155]
